# Systems analysis of phosphate-limitation-induced lipid accumulation by the oleaginous yeast *Rhodosporidium toruloides*

**DOI:** 10.1186/s13068-018-1134-8

**Published:** 2018-05-25

**Authors:** Yanan Wang, Sufang Zhang, Zhiwei Zhu, Hongwei Shen, Xinping Lin, Xiang Jin, Xiang Jiao, Zongbao Kent Zhao

**Affiliations:** 1 Division of Biotechnology, Dalian Institute of Chemical Physics, CAS, 457 Zhongshan Road, Dalian, 116023 People’s Republic of China; 2 Dalian National Laboratory for Clean Energy, Dalian Institute of Chemical Physics, CAS, 457 Zhongshan Road, Dalian, 116023 People’s Republic of China; 3 School of Food Science and Technology, Dalian Polytechnic University, Dalian, 116034 People’s Republic of China; 4 Beijing Bio-Fly Bioscience Co. Ltd., Beijing, 100080 People’s Republic of China

**Keywords:** *Rhodosporidium toruloides*, Microbial lipids, Oleaginous yeast, Phosphate-limitation, Systems biotechnology, Fatty acid biosynthesis, Triglycerides, Proteomics, Transcriptomics

## Abstract

**Background:**

Lipid accumulation by oleaginous microorganisms is of great scientific interest and biotechnological potential. While nitrogen limitation has been routinely employed, low-cost raw materials usually contain rich nitrogenous components, thus preventing from efficient lipid production. Inorganic phosphate (Pi) limitation has been found sufficient to promote conversion of sugars into lipids, yet the molecular basis of cellular response to Pi limitation and concurrent lipid accumulation remains elusive.

**Results:**

Here, we performed multi-omic analyses of the oleaginous yeast Rhodosporidium toruloides to shield lights on Pi-limitation-induced lipid accumulation. Samples were prepared under Pi-limited as well as Pi-repleted chemostat conditions, and subjected to analysis at the transcriptomic, proteomic, and metabolomic levels. In total, 7970 genes, 4212 proteins, and 123 metabolites were identified. Results showed that Pi limitation facilitates up-regulation of Pi-associated metabolism, RNA degradation, and triacylglycerol biosynthesis while down-regulation of ribosome biosynthesis and tricarboxylic acid cycle. Pi limitation leads to dephosphorylation of adenosine monophosphate and the allosteric activator of isocitrate dehydrogenase key to lipid biosynthesis. It was found that NADPH, the key cofactor for fatty acid biosynthesis, is limited due to reduced flux through the pentose phosphate pathway and transhydrogenation cycle and that this can be overcome by over-expression of an endogenous malic enzyme. These phenomena are found distinctive from those under nitrogen limitation.

**Conclusions:**

Our data suggest that Pi limitation activates Pi-related metabolism, RNA degradation, and TAG biosynthesis while inhibits ribosome biosynthesis and TCA cycle, leading to enhanced carbon fluxes into lipids. The information greatly enriches our understanding on microbial oleaginicity and Pi-related metabolism. Importantly, systems data may facilitate designing advanced cell factories for production of lipids and related oleochemicals.

**Electronic supplementary material:**

The online version of this article (10.1186/s13068-018-1134-8) contains supplementary material, which is available to authorized users.

## Background

A small number of microorganisms can accumulate lipids intracellularly to over 20% of dry cell weight under nutrient limitation [1], while lipid contents of many others are less than 10%. The conversion of carbohydrates and related materials into storage lipids by oleaginous microbes is of great scientific interest and biotechnological potential [2, 3]. In particular, microbial lipid production has been intensively studied because of its potential to supply alternative resources for functional lipids, renewable oleochemicals, and biodiesel [4]. It has been known that nitrogen-limitation can induce lipid accumulation, where cells down-regulate tricarboxylic acid (TCA) cycle and cell proliferation, but facilitate carbon flux for lipid biosynthesis [1, 5]. It should be noted that low-cost raw materials usually contain rich nitrogenous components, thus preventing from efficient lipid production. Limitation of other nutrients such as inorganic phosphate (Pi), sulfate, or iron has also been known to facilitate lipid production [6–8]. As Pi can be readily removed from aqueous streams at large scale, it is appealing to use Pi-limitation as a convenient strategy to explore nitrogen-rich raw materials for lipid production. Indeed, high cellular lipid contents were documented by different oleaginous hosts under Pi-limitation [8–10], even in the presence of excess nitrogenous nutrients [7, 11]. Nonetheless, the molecular basis of cellular responses to Pi-limitation and concurrent lipid accumulation by oleaginous species remains elusive.

Because phosphorus is an essential element for DNA, RNA, several ubiquitous cofactors, and phosphorylated proteins, Pi-limitation has major affects on cellular metabolism and physiology. By studying model microbes such as *Escherichia coli* [12], *Saccharomyces cerevisiae* [13, 14], and *Cryptococcus neoformans* [15], some fundamentals of cellular responses to Pi-limitation have been documented. Compared with glucose-limited condition, under Pi-limited aerobic condition, the budding yeast *S. cerevisiae* up-regulated 292 genes and activated a regulatory mechanism known as the PHO pathway, leading to an increased expression of multiple genes involved in Pi acquisition and uptake [16]. However, these model species did not accumulate lipids to a high content under Pi-limited conditions.

The red yeast *Rhodosporidium toruloides* (synonym *Rhodotorula toruloides*), a basidiomycetous fungus, can accumulate neutral lipids mainly as triacylglycerols (TAG) up to 70% of dry cell weight [1, 17]. More attractively, *R. toruloides* and other closely related yeasts can utilize some challenging substrates including xylose, biomass hydrolysates, waste glycerol and gas fermentation products [18, 19], and can co-produce valuable products such as carotenoids and useful enzymes [20]. In our previous studies, we completed genome annotation of *R. toruloides* np11 and did systems analysis of nitrogen-limitation-induced lipid production [5]. More recently, we also defined the lipid droplets (LD) proteome of this yeast and found tight association of LD with a highly expressed perilipin family protein RHTO_05627, and subsequently renamed it as lipid droplet protein 1 (Ldp1) [21].

The aim of this study was to gain insights at the systems level into the mechanism that *R. toruloides* contends with Pi-limitation and concurrently accumulates lipids. We compared the transcriptome, proteome, and metabolome of samples obtained under Pi-limited conditions with those under Pi-replete conditions and carried out confirmatory biochemical and genetic experiments. For the first time, we were able to integrate such data sets to delineate the molecular basis of cellular responses to Pi-limitation and establish key connections en route to lipid accumulation. Our results demonstrated that Pi-limitation facilitates up-regulation of phosphate metabolism, RNA degradation, and TAG biosynthesis, while down-regulation of ribosome biosynthesis and TCA cycle, leading to enhanced carbon flux for lipids. It was found that lipid production under Pi-limitation had unique mechanisms in terms of regulation of isocitrate dehydrogenation activity, NADPH supply, and diacylglycerol (DAG) metabolism. This study greatly enriched our understanding on microbial oleaginicity, cellular Pi metabolism, and their crosstalk. The information should be valuable to design advanced cell factories for production of lipid biosynthesis-derived chemical entities that are intensively pursued targets because of their diverse application potentials [22, 23].

## Methods

### Strain, media, and chemostat cultivations

The red yeast *R. toruloides* AS 2.1389 (China General Microbiological Culture Collection Center) was grown in 3-l stirred tank reactor (Baoxing Bio-Engineering Equipment, Shanghai, China) with a constant working volume of 2.0 l. The bioreactor was equipped with a 405-DPAS-SC-K8S/225 pH electrode and an InPro 6800 O_2_ sensor (Mettler Toledo).

The seed medium contained (g/l): glucose·H_2_O 30, (NH_4_)_2_SO_4_ 5, Na_2_HPO_4_ 1, KH_2_PO_4_ 1, and MgSO_4_ 1.5, pH 6.0. The Pi-limited lipid production medium contained (g/l): glucose·H_2_O 30, (NH_4_)_2_SO_4_ 5, Na_2_SO_4_ 0.94, Na_2_HPO_4_ 0.06, K_2_SO_4_ 0.64, and MgSO_4_ 1.5, pH 6.0 with a C/P molar ratio of 8000 and the Pi-replete medium (g/l): glucose·H_2_O 30, (NH_4_)_2_SO_4_ 5, Na_2_HPO_4_ 1, KH_2_PO_4_ 1, and MgSO_4_ 1.5, pH 6.0. After being sterilized at 121 °C for 15 min, the medium was supplemented with a 100-fold diluted trace element solution [17]. The trace element solution contained per liter: CaCl_2_·2H_2_O 4.0 g, FeSO_4_·7H_2_O 0.55 g, ZnSO_4_·7H_2_O 0.10 g, MnSO_4_·H_2_O 0.076 g, and 100 μl of 18 M H_2_SO_4_, and was sterilized at 121 °C for 15 min.

The seed culture was done at 30 °C in 250-ml Erlenmeyer flask on a rotary shaker at 200 rpm for 28 h. The chemostat culture was initiated by adding 200 ml of the seed culture into 1.8-l of sterilized lipid production medium and held at 30 °C, pH of 5.6 (maintained by automatic addition of 2 M NaOH) with a stirrer speed of 500 rpm and a dissolved oxygen above 85% of air saturation. The airflow was 100 l/h. The dilution rate was 0.085 and 0.3 h^−1^ for the culture feeding with the Pi-limited medium and the Pi-replete medium, and the samples were designated as *P*0 and *F*3, respectively. Steady-state samples were taken after five volume changes. The fermentation media used in this work are listed in Table 1.

**Table 1:** Parameters and results of chemostat culture of *R. toruloides*

Culture code	*F*3 (Pi-replete)	*P*0 (Pi-limited)
Feeding nutrients		
Glucose (g/l)	27	27
PO_4_^3−^ (mM)	10.14	0.11
C/P molar ratio	63	2164
Dilution rate (h^−1^)	0.3	0.085
Effluent nutrients		
Glucose (g/l)	22.3 ± 0.6^a^	22.3 ± 0.6^a^
PO_4_^3−^ (mM)	7.35 ± 0.25^a^	ND
Production parameters		
Cell mass (g/l)	0.30 ± 0.07^a^	0.83 ± 0.06^a^
Lipid content (%)	7.3 ± 2.3^a^	43.9 ± 0.5^a^
*Y*_X,S_ (g cell mass per g glucose)	0.06 ± 0.01^a^	0.18 ± 0.01^a^
*Y*_P,S_ (mg oil per g glucose)	4.5 ± 1.4^a^	88.3 ± 5.8^a^
*Y*_X,Pi_ (g cell mass per g PO_4_^3−^)	1.1 ± 0.2^a^	79.4 ± 6.7^a^
*Y*_P,Pi_ (g oil per g PO_4_^3−^)	0.08 ± 0.02^a^	34.8 ± 2.1^a^

*F*3, medium with abundant phosphate with dilution rate of 0.3 h^**−1**^; *P*0, medium with trivial amount of phosphate with dilution rate of 0.085 h^−1^; ND, not detectable

^a^Average ± s.d. of three biological samples

### Total sugar content and lipid content

Glucose was measured using an SBA-40D glucose analyzer (Shandong Academy of Sciences, Jinan, China). Cells from 30-ml culture broth were harvested by centrifugation at 8000*g* for 5 min and washed twice with distilled water. Cell mass, expressed as dry cell weight, was determined gravimetrically after drying the wet cells at 105 °C for 24 h.

Dried yeast cells were digested with 4 M HCl at 78 °C for 1 h before extraction with chloroform/methanol (1:1, vol/vol). The extracts were washed with 0.1% NaCl, dried over anhydrous Na_2_SO_4_, and evaporated in vacuo, and the residue was dried at 105 °C for 24 h to give the total lipid [17]. Lipid content was expressed as gram lipid per gram DCW. The lipid content was calculated as gram lipid produced per gram sugar consumed and the results are listed in Table 1.

### RNA sampling and isolation

Samples for RNA isolation from chemostat cultures were taken by rapidly sampling 40 ml of culture into a tube with ice to decrease the sample temperature. Cells were then centrifuged (8000 rpm at 4 °C for 2 min) and instantly stored at − 80 °C until further use. Total RNA was extracted from samples using RNAiso Plus (Takara, Dalian, China) in accordance with the manufacturer’s instructions and samples were stored at − 80 °C. The RNA concentration and quality were tested using the SMA1000 UV spectrophotometer (Merinton, Beijing, China).

### Library preparation and sequencing

For library preparation, the mRNA was enriched using the oligo(dT) magnetic beads, mixed with the fragmentation buffer, and fragmented into short fragments (about 200 bp). Then, the first strand of cDNA was synthesized using random hexamer primer. Buffer, dNTPs, RNase H, and DNA polymerase I were added to synthesize the second strand. The double-strand cDNA was purified with magnetic beads. End repair and 3′-end single-nucleotide A (adenine) addition was then performed. Finally, sequencing adaptors were ligated to the fragments. The fragments were enriched by PCR amplification. During the quality control step, Agilent 2100 Bioanaylzer and ABI Step One Plus Real-Time PCR System were used to qualify and quantify of the sample library. The library products were ready for sequencing via Illumina HiSeq™ 2000 platform.

### Mapping reads to the reference genome

As the raw reads which were transferred from base calling may contain low-quality reads and/or adaptor sequences, preprocessing is necessary before starting further analysis [24, 25]. Clean reads were mapped to *R. toruloides* NP11 and *R. toruloides* ATCC 204091 combined reference gene sequences [5, 26] and to *R. toruloides* reference genome sequences set using SOAPaligner/SOAP2 [27]. Not more than two mismatches were allowed in the alignment.

### Gene expression analysis

The expression level for each gene was determined by the numbers of reads uniquely mapped to the specific gene and the total number of uniquely mapped reads in the sample. The gene expression level was then calculated using the reads per kilobases per million reads (RPKM) method [28], which quantified transcript levels in reads per kilobase of exon model per million mapped reads and computed RPKM by software package Cufflinks. The statistical significance of the differential expression of each gene was determined using the *P* value. The FDR (false discovery rate) ≤ 0.001 and |log_2_ Ratio| ≥ 1 were used to identify differentially expressed genes (DEGs) [29]. The *P* value and FDR were acquired by a more commonly used software package DEseq.

### Gene ontology and pathway enrichment analysis

Gene ontology (GO) is an international standardized gene functional classification system which offers a dynamic-updated-controlled vocabulary and strictly defined concepts to comprehensively describe the properties of genes and their products in any organism [30, 31]. GO contains cellular component, molecular function, and biological process domains. GO enrichment analysis provides all GO terms that are significantly enriched in DEGs compared with the genome background, and filters the DEGs that correspond to biological functions. With nr annotation, the Blast2GO program was used to obtain the GO annotation of the DEGs. WEGO software [32] was then used to perform GO functional classification for DEGs and determine the distribution of gene functions of the species at the macrolevel. The Kyoto Encyclopedia of Genes and Genomes (KEGG) is the major public pathway-related database [33]. Pathway enrichment analysis identifies significantly enriched metabolic pathways or signal transduction pathways in DEGs compared with the whole genome background. A *Q* value ≤ 0.05 was defined as a significantly enriched pathway in terms of DEGs.

### Protein extraction, digestion, and labeling with iTRAQ reagents

For cell lysis and protein extraction, all steps were carried out on ice to avoid denaturation of proteins. Yeast cells were collected and washed as previously. Lysis buffer containing the following compositions gave the highest and most stable protein level when treating the same amount and batch of cells: 8-M Urea, 50-mM DTT, 50-mM Tris–Cl (pH7.6), 100-mM NaCl, 0.1% Triton X-100, 1-mM EDTA, and 1-mM PMSF. The pH value of lysis buffer was adjusted to 7.4 using 1-M HCl. Three hundred microliter lysis buffer and approximate 200 µl glass beads were added to the pellet and disruption was conducted in the homogenizer by shaking ten periods of 30 s with 2-min cooling intervals on ice. Cell lysates were centrifuged at 8000 rpm for 10 min at 4 °C and supernatant was transferred to clean tubes and stored at − 80 °C.

Prior to further treatment, lipid contaminants in protein extract were removed by organic solvents according as demonstrated [34]. Protein concentration was determined using Quick Start Bradford Protein Assay (Bio-Rad) according to Bradford method [35] followed by precipitation by 2-D Clean-Up Kit (GE Healthcare). Sample preparation and iTRAQ labeling was carried out using an iTRAQ 8-plex kit from AB Sciex. Briefly, protein pellets were re-dissolved in 20-µl 0.5-M triethylammonium bicarbonate and 1-µl 2% SDS. Afterwards, 2-µl 50-mM tris(2-carboxyethyl)phosphine was added to reduce the disulfide bonds of the proteins at 60 °C for 1 h. Alkylation was carried out by adding 1-µl 200-mM methyl methanethiosulfonate to reversibly block cysteine group at room temperature for 15 min. Digestion of each sample was then processed at 37 °C for 16 h with a final concentration of 10-ng/µl sequencing grade-modified trypsin solution (AB Sciex). The digested peptides were labeled with the iTRAQ tags (AB Sciex) as follows: Rt-1, iTRAQ 113; Rt-2, iTRAQ 114; *F*3-1, iTRAQ 115; *F*3-2, iTRAQ 116; *F*3-3, iTRAQ 117; *P*0-1, iTRAQ 118; *P*0-2, iTRAQ 119; *P*0-3, iTRAQ 121. Among them, Rt-1 and Rt-2 were independent samples of *R. toruloides* cells cultured under Pi-replete conditions. The labeled samples were then combined together accordingly and desalted by C18 Cation Exchange Cartridge system (AB Sciex) together with C18 Sep-Pak Plus Short Column (55–105 µm) (Waters) to clean up mixture and remove salt and contaminants. The samples were then lyophilized and dissolved with 5% acetonitrile with 0.1% formic acid to proper concentration, and subjected to 2-D Nanoflow LC–MS/MS as described [36]. The collected peptides were detected by 5600 Triple TOF Analyzer (AB Sciex). A maximum of 30 precursors per cycle from each MS spectrum were selected for MS/MS analysis. Tandem mass spectrum was recorded in high-sensitivity mode with rolling collision energy on and iTRAQ reagent collision energy adjustment on.

### Data analysis and interpretation of protein identification

Protein identification and relative iTRAQ quantification were performed with ProteinPilot software 5.0 (AB Sciex) using the Paragon algorithm for the peptide identification which was further processed by Pro Group algorithm where isoform specific quantification was adopted to trace the differences between expressions of various isoforms. The complete set of raw data files of each run was searched against the annotated genome including 8171 proteins [5] using the Paragon and Pro Group algorithms (AB Sciex). For iTRAQ quantification, the peptide was automatically selected by Pro Group™ algorithm to calculate the reporter peak area, error factor (EF), and *P* value. The resulting data set was auto bias-corrected to get rid of any variations imparted due to the unequal mixing during the combination of different labeled samples. This software counts each modified peptide as a unique one. The peak areas are extracted from the database by ProteinPilot to process the raw data to yield quantification data. The intensities of the reporter ions were obtained from each spectrum. The quantification was obtained from the normalized data, which were the total intensities of all peptide fragments for each protein. In addition, a reverse database search strategy was also adopted to estimate the false discovery rate (FDR) for peptide identification. In this study, only peptides identified with confidence interval values of no less than 95% (unused confidence threshold ProtScore > 1.3) were reported, and the EF < 2, *P* < 0.05, corresponding to FDR < 5% were used for protein identification compilation and subsequent quantitation calculation. The relative abundance of proteins was calculated based on individual peptide ratios from three experimental replicates, and the relative values for protein differential expression were presented as mean ± SD. The results were then exported into Microsoft Excel for manual data interpretation. The meaningful cut-off value with a 1.5 fold-change was used to designate significant differences in protein expression among the Pi-limited group and the Pi-replete group. Subsequently, the up-regulation and down-regulation of proteins was finalized.

### Metabolomic analysis procedure

The five parallel samples of each experimental group were harvested as previously and methanol was used to extract metabolites [37]. Cells were rapidly freeze-dried at a low temperature. After that, 800 ml of methanol was added to 100 mg of the cells and then extracted by ultrasound for 30 min. The extract solution was centrifuged at 12,000*g* for 10 min. After extraction, samples were determined by LC-Q/TOF–MS (Agilent, 1290 Infinity LC, 6530 UHD and Accurate-Mass Q-TOF/MS). An UPLC C18 column (100 × 2.1 mm, ø 1.8 μm, Agilent Technologies) was used to perform the chromatographic separation and the C18 column temperature was maintained at 40 °C. The mobile phase consisted of 0.1% formic acid in water (A) and 0.1% formic acid in acetonitrile (B). The flow rate was 0.4 ml/min. The gradient was applied as follows: *t* = 0 min, 95% A and 5% B; *t* = 2 min, 95% A and 5% B; *t* = 17 min, 5% A and 95% B; *t* = 19 min, 5% A and 95% B.

The mass spectrometer was operated in both positive and negative ion modes and set to the total ion chromatogram (TIC) mode. Operating conditions of positive ions were as follows: capillary voltage, 4 kV; sampling cone, 35 kV; source temperature, 100 °C; desolvation temperature, 350 °C; cone gas flow, 50 l/h; desolvation gas flow, 600 l/h. The conditions of negative ions were as follows: capillary voltage, 3.5 kV; sampling cone, 50 kV; source temperature, 100 °C; desolvation temperature, 300 °C; cone gas flow, 50 l/h; desolvation gas flow, 700 l/h. To ensure that the mass was measured accurately [38], leucine-enkephalin was used as a reference lock-mass compound at a concentration of 2 ng/μl and a flow rate of 5 μl/min. In both positive and negative modes, leucine-enkephalin was detected at 556.2771 and 554.2615 Da, as [M+H]^+^ and [M−H]^−^ ions, respectively.

### Metabolomic data analysis and interpretation

The metabolomics data were extracted and pre-processed by the Mass Profiler software developed by Agilent and edited in the EXCEL 2007 software to organize the final results into a two-dimensional data matrix, including variables (rt/mz, i.e., retention time/mass ratio), observed quantity (sample) and peak intensity. All data were then normalized to total signal integration, and the edited data matrix was imported into Simca-P software (version 11.5), which provides effective algorithms for principal component analysis (PCA), partial least-squares-discriminant analysis (PLS-DA) and the orthogonal partial least-squares-discriminant analysis (OPLS-DA).

We used the VIP (variable importance in the projection) value (threshold > 1) of the OPLS-DA model and the *P* value (threshold < 0.05) of the *t* test to find the differential metabolites. The qualitative method for metabolites was to search the online database (http://metlin.scripps.edu/) (compare mass-to-mass ratio *m*/*z* or exact molecular mass). The meaningful cut-off value with a 1.2 fold-change was used to designate significant differences in metabolites among the Pi-limited group and the Pi-replete group.

### Real-time quantitative PCR analysis

RT-qPCR analysis was used to verify the DGE results. The RNA samples were the same as for the DGE experiments. Gene-specific primers were designed according to the reference unigene sequences using Primer Express 3.0 software for RT-qPCR analysis (Additional file 1: Table S1). RT-qPCR was performed according to the TaKaRa manufacturer specifications (TaKaRa SYBR^®^ PrimeScript™ RT-qPCR Kit, Dalian, China) and performed on an Eco Real-Time PCR System (Illumina, USA). RT-qPCR was conducted in duplicate for each gene. The reaction mixture contained 5 μl SYBR Green (TaKaRa), 1 μl each of forward and reverse primers (2 μM), 1 μl template DNA, and nuclease-free water diluted to a final volume of 10 μl. RT-qPCR conditions were as follows: 95 °C for 30 s, (95 °C for 5 s, 60 °C for 30 s) for 40 cycles, 95 °C for 15 s, 55 °C for 15 s, 95 °C for 15 s. To ensure the specificity of the RT-qPCR products, melting curves of the amplifications were checked after thermal cycling. GAPDH was used as a normalizer, and the relative expression levels of genes were presented by 2^−∆∆CT^ method [39].

### Partial purification of recombinant NAD-dependent IDH

Total RNA was isolated from *R. toruloides* AS 2.1389 as described [40]. The full-length cDNA fragments of IDH1 and IDH2 were obtained previously [41] and ligated with pMD18-T, resulting in T-IDH1 and T-IDH2. The full-length cDNA fragments coding Idh1 (RHTO_01289) and Idh2 (RHTO_01290) were individually cloned into the plasmid pET28a by RF cloning method, and the plasmids including Idh1 and Idh2 were transformed into *E. coli* BL21(DE3). Recombinant Idh1 and Idh2 were produced upon isopropyl-β-d-thiogalactoside induction, purified by Ni-affinity chromatography, and analyzed by SDS-PAGE gel scan analysis. Activity assays were done with a 1:1 molar ratio of Idh1 and Idh2 mixture in triplicates.

### Over-expressing ME1 in *R. toruloides* AS 2.1389

All the binary vectors were constructed based on the pZPK plasmid, which could be selected in both *E. coli* and *Agrobacterium tumefaciens AGL1* with kanamycin [42]. The pZPK-pPGK-HYG-Tnos-pPHO89-MCS-Thsp vector [43] harbored a PGK1 and PHO89 promoter, the nos and hsp terminator, respectively, hygromycin marker and MCS contains three endonuclease restriction sites *Eco*RV, *Nco*I, and *Spe*I. The gene *ME1* (Gene ID: 27367808) was PCR amplified from the cDNA of *R. toruloides* and the PCR products were purified, digested by *Nco*I and *Spe*I, and ligated with the vector pZPK-pPGK-HYG-Tnos-pPHO89-MCS-Thsp digested by the same pair enzymes. The ligation mixtures were used to transform *E. coli* DH5α competent cells and the transformants were selected on LB plates supplemented with 50-μg/ml kanamycin. Positive colonies were further identified by colony PCR, and then, the plasmids were extracted and verified by DNA sequencing. The correct plasmids were transformed into *A. tumefaciens* AGL1 by electroporation and strains were selected on LB plates supplemented with 50 μg/ml kanamycin. Transformants were confirmed by colony PCR. Transformation of *R. toruloides* was done according to a published method [42].

### Lipid production experiment

Lipid production experiments of the strains over-expressing Me1 were done in duplicates at 30 °C for 120 h in 250-ml flasks on a rotary shaker at 200 rpm. Cultures were initiated upon 45 ml of the lipid production medium inoculated with 5 ml of 28-h-old seed culture (the same as chemostat cultivations). The lipid production medium had the same C/P ratio as chemostat cultivations, but the glucose content was 50 g/l. Cells in 30 ml of culture broth were harvested by centrifugation, washed twice with distilled water, and then dried at 105 °C for 24 h to obtain dry cell weight (DCW). Cellular lipid was extracted with chloroform–methanol [17]. Lipid content was calculated as gram lipid per gram and then multiplied by 100%.

## Results

### Lipid production under Pi-replete and Pi-limited chemostat conditions

To prepare reliable samples representing cellular performance under Pi-replete and Pi-limited conditions, we grew chemostat cultures of *R. toruloides* cells using minimal medium with 27 g/l glucose but different Pi loadings to ensure the initial carbon-to-phosphorus molar ratio (C/P) of 63 and 2164, and samples were coded as *F*3 and *P*0, respectively. Results showed that both effluents contained identical residual glucose, while Pi dropped to below detection limit for the Pi-limited (*P*0) culture (Table 1). Cellular lipid contents for the Pi-replete and Pi-limited samples were 7.3 and 43.9%, respectively. Furthermore, under Pi-limitation, the cell mass yield on glucose (*Y*_X,S_) and lipid yield (*Y*_P,S_) of 180 and 88.3 mg/g, respectively, were 3.0- and 18.6-fold higher than those under Pi-replete conditions. These results showed that cells had lipids less that 10% without nutrient limitation, while cells accumulated lipids to over 40% under Pi-limitation. Our data were in agreement with a number of earlier observations that Pi-limitation facilitated lipid production by oleaginous fungi [6, 8–10]. More interestingly, the much greater increase in lipid yield than in cell mass yield suggested that cells used glucose more efficiently for lipid biosynthesis under Pi-limitation. Concurrently, the apparent cell mass yield on Pi (*Y*_X,Pi_) and lipid yield (*Y*_P,Pi_) were 72- and 435-fold, respectively, higher under Pi-limitation (Table 1), indicating that cells turned over Pi much more efficiently for metabolic activities.

### RNA-seq and digital gene expression analysis

To illuminate the transcriptome response to Pi-limitation by *R. toruloides*, we performed high-throughput RNA-seq and digital gene expression analysis of those samples obtained under chemostat conditions. Total RNA samples were extracted, and mRNA was enriched using the oligo(dT) magnetic beads and sheared into short fragments. These mRNAs of about 200 bases were used as templates for cDNA synthesis. The cDNAs were then PCR amplified and sequenced using an Illumina HiSeq™ 2000 platform. There were approximately 11 million raw reads per library for quantitative analysis of gene expression. Of those reads, 54.42 and 51.36% from the Pi-replete and Pi-limited samples, respectively, mapped to *R. toruloides* np11 reference genes, and slightly more reads mapped to the genome (Additional file 2: Table S2). After filtering the adaptor sequences, regions containing N sequences and low-quality sequences, there were still over 10 million clean reads in each RNA-Seq library. The percentages of clean reads among the raw reads reached over 95% in both libraries, indicating high-quality original data (Additional file 2: Table S2).

**Fig. 1:**
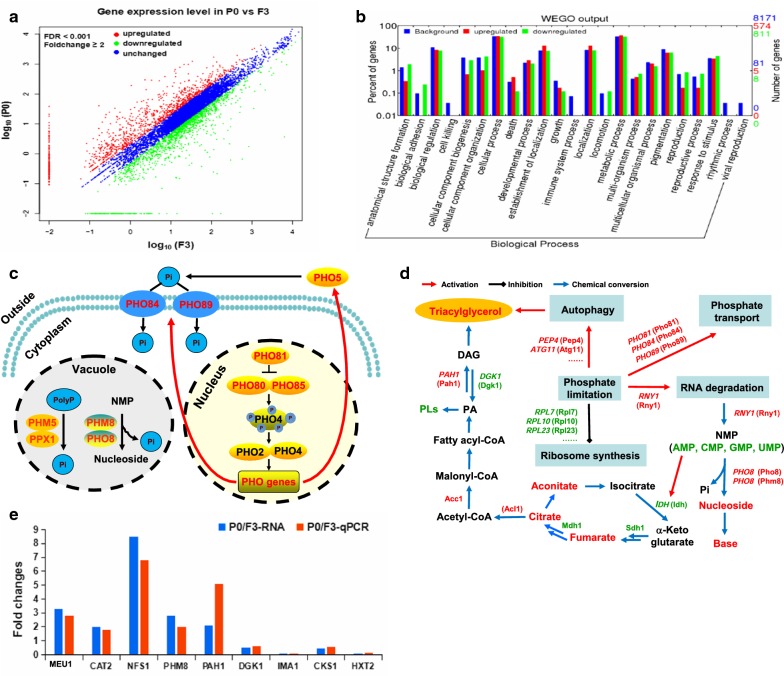
Differential transcriptomic analysis of *R. toruloides* in response to phosphate-limitation. **a** Global profiling of gene expression changes in *P*0 vs *F*3. **b** Gene ontology enrichment analysis of differentially expressed genes. GO terms of biological process (level 3) were analyzed and enriched. **c** Genes being up-regulated shown in red for Pi-related metabolism. **d** Multi-omic responses to Pi-limitation and lipid accumulation. The red indicated up-regulated gene, protein, or enriched metabolite, while the green indicated down-regulated gene, protein, or lowered metabolite. **e** Comparison of transcription levels of representative genes between RNA-Seq data and RT-qPCR results

In total, there were 7970 genes observed in this study (Additional file 3: Table S3). Of 7797 and 7868 genes that were detected in the Pi-replete and Pi-limited sample, respectively; many genes were differentially expressed (Fig. 1a). Compared with the Pi-replete sample, the Pi-limited samples had 585 and 817 genes being up- and down-regulated, respectively. Those differentially regulated genes are involved in a number of biological processes including Pi-related metabolism, ribosome biogenesis, RNA degradation, and response to stimulus (Fig. 1b). Specifically, gene encoding H^+^/Pi-symporter *PHO84* (RHTO_06822), Na^+^/Pi-symporter *PHO89* (RHTO_01284), and the cyclin-dependent kinase (CDK) inhibitor *PHO81* (RHTO_02991), were all significantly up-regulated (Fig. 1c). Besides, the polyphosphate (polyP) degradation enzymes *PHM5* (RHTO_03914) and *PPX1* (RHTO_07023), and the secretory phosphatases Pho5 (RHTO_04431), were slightly up-regulated (Fig. 1c). In general, the biological consequences of such changes should lead to activating Pi-associated metabolism and increasing Pi supply. The down-regulated genes were related to ribonucleoprotein complex biogenesis. At the transcription level, 94 of the 111 genes being annotated as ribosome structural components were down-regulated. This suggested that, under Pi-limitation, ribosome synthesis was suppressed. During ribosome degradation, not only ribosomal proteins, but also a large amount of ribosomal RNAs should be degraded in the vacuole/lysosome. Indeed, *RNY1* (RHTO_05069), the gene encoding an endonuclease with weak nucleobase specificity was 65.5-fold up-regulated, and two nucleotidase-encoding genes, *PHM8* (RHTO_07849) and *PHO8* (RHTO_03053), were up-regulated by 2.8- and 80-fold, respectively. It should be noted that up-regulation of *PHM8* was confirmed by RT-qPCR analysis (Fig. 1e). It is known that these nucleosides may be further converted to bases by endogenous hydrolyases [44]. Thus, our data suggested that Pi-limitation induces degradation of RNA and nucleotides and activation of pathways to source more Pi. Meanwhile, Pi consumption process is inhibited such as ribosome biosynthesis.

To get more insights on lipid accumulation under Pi-limitation, we analyzed the changes of those genes related to central metabolism and lipid biosynthesis. There were significant transcriptional differences for genes involved in the late stage of TAG biosynthesis. Specifically, phosphatidate phosphatase (Pah) gene *PAH1* (RHTO_04894) was 2.1-fold up-regulated, while DAG kinase gene *DGK1* (RHTO_06970) was 2.1-fold down-regulated, leading to enhanced DAG formation and reduced consumption of DAG for phospholipids synthesis, respectively (Fig. 1d). It should be noted that up-regulation of *PAH1* and down-regulation of *DGK1* were confirmed by RT-qPCR analysis (Fig. 1e). Interestingly, no significant change was observed for those genes involved in lipid degradation (i.e., lipolysis and the β-oxidation cycle), indicating that lipid accumulation was not a resulted of down-regulation of lipid degradation. However, under nitrogen-limitation, a number of genes related to lipid degradation were up-regulated [5]. In terms of those 226 genes encoding proteins associated with LD [21], there were 80 genes up-regulated under Pi-limitation. Notably, the transcription level of *LDP1* (RHTO_05627) was up-regulated by 40-fold in this study, while it was up-regulated by 20-fold under nitrogen-limitation [5].

In *R. toruloides*, 32 genes associated with autophagy were identified [5]. It should be noted that about 25% of those genes associated with autophagy were up-regulated under Pi-limitation. These included *ATG1* (RHTO_06351), *ATG11* (RHTO_00852), *ATG14* (RHTO_05412), *ATG15* (RHTO_00361), and *PEP4* (RHTO_07813). Autophagy has been suggested to make more room for lipid accumulation through the clearance of other organelles or portions thereof. In fact, up-regulation of the autophagy process was demonstrated under nitrogen-limited lipid production [5].

### Comparative proteomic analysis

**Fig. 2:**
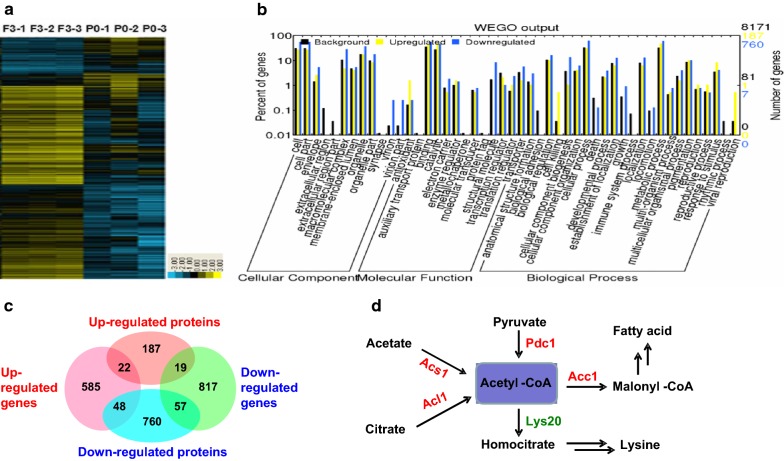
Comparative proteomic analyses of *R. toruloides* samples prepared under Pi-limited (*P*0) and Pi-replete (*F*3) conditions. **a** Heatmap of up- and down-regulated proteins in *P*0 and *F*3 compared with those of the control sample Rt1. **b** Gene ontology enrichment analysis of up- and down-regulated proteins. **c** Venn diagram of up- and down-regulated genes and proteins in *P*0 vs *F*3. **d** Up- and down-regulated proteins related to acetyl-CoA metabolism

Total proteins of both Pi-replete and Pi-limited samples were extracted, digested by sequencing grade trypsin, labeled with the isobaric tags for relative and absolute quantitation (iTRAQ) tags, and subjected to shotgun liquid chromatography (LC)–MS analysis. Each MS/MS spectrum was searched against the *R. toruloides* proteome database being generated locally. A total of 73815 unique peptides were identified. These peptides could be mapped to 4212 of *R. toruloides* protein models (Additional file 4: Table S4), representing 51.5% of the annotated coding sequences. Further analysis using a stringent criterion (i.e., differential of quantitative information based on peptides identified to each protein was below 2) led to identification of 2438 proteins with high quantification confidence. The heatmap of comparative protein distribution is shown in Fig. 2a. Compared to the Pi-replete sample, the Pi-limited sample had 187 proteins up-regulated and 760 down-regulated. Gene ontology term enrichment indicated that proteins associated with lipid metabolism and reproductive process were up-regulated, while translation, transport and structural molecule-associated proteins were down-regulated (Fig. 2b), suggesting that the protein biosynthesis machinery was suppressed. While there were major differences between differential transcriptomic and proteomic data (Fig. 2c), proteins related to the PHO system, including Pho81, Pho84, and Pho89, were up-regulated (Fig. 2b). These data were in good agreement with the transcriptomic data (Additional file 3: Table S3). We also found up-regulation of Pho8 and Phm8, two proteins related to nucleotide degradation, which would lead to enhanced formation of nucleosides and bases. Thus, both transcriptomic and proteomic data suggested that *R. toruloides* cells activated processes to source Pi under Pi-limitation.

There were 30 proteins involved in central/lipid metabolism found at elevated levels under Pi-limitation, namely, *P*0/*F*3 value higher than 1.2. For example, the LD structural protein Ldp1 had 17.8-fold higher level in the Pi-limited samples, which was consistent with the up-regulation at the transcription level and its potential biological function. Fatty acid biosynthesis-associated protein acetyl-CoA carboxylase (Acc1, RHTO_02004) and 3-hydroxyacyl-CoA dehyrogenase (Hcd1, RHTO_05520) was 3.0- and 3.3-fold, respectively, up-regulated. Interestingly, over-expression of native Acc1 has been shown to improve lipid production by a *R. toruloides* strain [45]. Three enzymes, ATP-citrate lyase (Acl1, RHTO_03915), acetyl-CoA synthase (Acs1, RHTO_08027), and pyruvate decarboxylase (Pdc1, RHTO_00098), were up-regulated by 1.9-, 2.0-, and 3.3-fold, for enhanced conversion of citrate, acetate and pyruvate, respectively, into acetyl-CoA, the common intermediate for fatty acid biosynthesis. It was also noteworthy that homocitrate synthase (Lys20, RHTO_06979) level in the Pi-limited samples was only 13% that of the Pi-replete samples, this should lead to a reduced acetyl-CoA consumption (Fig. 2d).

The levels of proteins associated with the TCA cycle, including Idh1 (RHTO_01289), Sdh1 (RHTO_05714), Sdh2 (RHTO_06068), and Mdh1 (RHTO_04363), decreased in the Pi-limited sample, namely, *P*0/*F*3 value below 0.8, indicating that the TCA cycle activity was reduced under Pi-limitation. In fungi, the transhydrogenation machinery, composed of pyruvate carboxylase (Pyc), NADP^+^-dependent malic enzyme (Me), and malate dehydrogenase, can convert NADH to NADPH [1]. It was found that both Pyc1 and Me1 had reduced levels, suggesting that NADPH formation through transhydrogenation was suppressed under Pi-limitation. Interestingly, however, the transcription of *ME1* (RHTO_03795) was up-regulated by 3.6-fold, suggesting that malic enzyme activity was regulated at different levels.

### Metabolomic analysis

To find more insights at the molecular level into Pi-related metabolism and lipid accumulation, we analyzed the metabolome of samples using an LC-Q/TOF MS-based approach. Cells were rapidly freeze-dried and the metabolites were extracted by cold methanol. Total ion chromatograms of quality control (QC) samples are shown in Additional file 7: Figure S1a and b. To test whether there were metabolic differences between the Pi-limited group and the Pi-replete group, we performed principal components analysis (PCA) with biological sextuple. Two principal components were obtained under positive and negative ion models, and the score plots are shown in Additional file 7: Figures S2 and S3. As the *R*^2^*X* values were both higher than 0.4 (Additional file 5: Table S5), the non-supervised model suggested the presence of metabolic differences between the two groups. We further adopted partial least-squares-discriminant analysis (PLS-DA) and the orthogonal partial least-squares-discriminant analysis (OPLS-DA). One principal component was identified under both positive and negative ion models, and the PLS-DA and OPLS-DA modeling of *P*0 vs *F*3 are shown in Additional file 7: Figures S2 and S3. Data for quality control of the modeling process are shown in Additional file 5: Table S5. In PLS-DA, the score of *Q*^2^ in *P*0 vs *F*3 was higher than 0.89, suggesting that the model was believable. In OPLS-DA, the result of *Q*^2^ higher than 0.5 in *P*0 vs *F*3 indicated that the model was authentic.

We identified 123 metabolites of which concentrations had significant changes between the two groups. Specifically, 63 metabolites had higher concentrations and 60 had lower contents in the Pi-limited group (Additional file 6: Table S6). A few nucleosides and bases derivatives exhibited much higher concentrations in the Pi-limited samples (Additional file 7: Figure S1c). These results thus provide direct evidences to correlate up-regulation of degradation of RNA and nucleotides at the transcriptional and translational levels (*vide ante*).

**Fig. 3:**
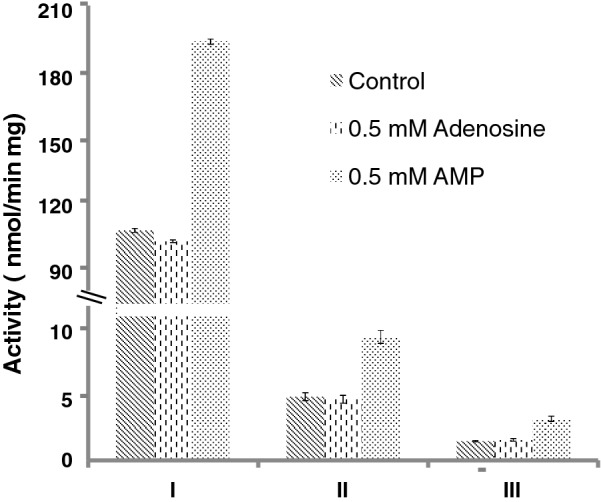
Effects of adenine derivatives on the activity of NAD-specific Idh. (I) Recombinant Idh1 and Idh2 at 1:1 molar ratio; (II) Crude cell extracts from cells under Pi-replete conditions; (III) Crude cell extracts from cells under Pi-limited conditions. All assays were performed in triplicates

Three TCA cycle intermediates were found. Aconitate, citrate, and fumarate had 2.5-, 2.2-, and 1.7-fold, respectively, higher level in the Pi-limited sample. One explanation for higher contents of aconitate and citrate was that the activity of a downstream reaction decreased. In oleaginous yeasts, it has been known that the downstream enzyme isocitrate dehydrogenase (Idh) requires adenosine monophosphate (AMP) for full activity [1]. To test whether Idh can be activated by AMP, we expressed *R. toruloides* Idh in *E. coli* BL21 (DE3) and purified the recombinant protein to homogeneity. Activity assays indicated that Idh had roughly twofold higher activities in the presence of AMP, and that adenosine had no such activating effects (Fig. 3). Moreover, crude cell extracts of *R. toruloides* cells cultivated under either Pi-limited or Pi-replete conditions also showed similar Idh activity profiles. These biochemical data confirmed that Idh can, indeed, be activated by AMP. Under Pi-limitation, cellular level of AMP was low due to up-regulation of nucleotide degradation enzymes, Pho8 and Phm8, thus leading to reduced Idh activity. Therefore, the flux of isocitrate to oxalosuccinate was reduced, resulting in accumulation of aconitate and citrate.

More importantly, the Pi-limited samples had 170,000- and 180,000-fold lower contents of PE (18:2) and PC (16:0), respectively, than the Pi-replete samples, indicating that phospholipids synthesis was reduced under Pi-limitation. These data were in agreement with transcriptional up-regulation of phosphatidate phosphatase (*PAH1*) while down-regulation of *DGK1*. Reduced formation of phospholipids may provide more DAG for TAG production.

Notably, the Pi-limited sample had 57,000-fold lower NADPH content than the Pi-replete sample. The contents of phosphogluconate, ribose 5-phosphate, and sedoheptulose 7-phosphate, three intermediates involved in the pentose phosphate (PP) pathway, were 190,000-, 390,000- and 6,3000,000-fold, respectively, lower in the Pi-limited sample. Albeit there was no apparent down-regulation of genes or enzymes associated with the PP pathway, drastically reduced concentrations of metabolic intermediates suggested a lower pathway activity. This should lead to less NADPH production as the PP pathway can generate NADPH. In addition, down-regulation of Me1 could further reduce NADPH formation through the transhydrogenation cycle, also known as the pyruvate–oxaloacetate–malate cycle. These results suggested that lipid production may be improved which should more NADPH be produced to fuel fatty acid biosynthesis. To test this idea, we constructed a cassette ensuring over-expression of Me1 and transformed *R. toruloides* AS 2.1389 cells using the *Agrobacterium*-mediated transformation approach [42]. Results showed that the engineered strain had 1.8-fold higher lipid content, and produced 1.9-fold more lipids, than the parent strain did under identical culture conditions in the Pi-limited medium (Table 2). Interestingly, it has been shown that over-expression of Me1 in *R. toruloides* IFO0880 led to only minor (24%) lipid titer increase in low-nitrogen media [46].

**Table 2:** Results of lipid production by wild-type *R. toruloides* and the engineered strain *R. toruloides*-Me1

Sample ID	DCW (g/l)	Lipid content (wt%)	Lipid (g/l)	Relative fatty acid content (%)					
				C14:0	C16:0	C16:1	C18:0	C18:1	C18:2
*R. toruloides*	9.8 ± 0.2	32 ± 1	3.2 ± 0.1	1.9 ± 0.1	23.8 ± 0.6	0.5 ± 0.0	10.6 ± 0.3	58.6 ± 0.3	4.6 ± 0.4
*R. toruloides*-Me1	10.3 ± 0.6	59 ± 5	6.1 ± 0.2	1.7 ± 0.3	21.9 ± 2.0	0.2 ± 0.2	15.6 ± 0.4	56.8 ± 0.7	3.7 ± 0.6

Cells were cultivated with 50 g/l glucose at an initial C/P molar ratio of 2159

## Discussion and conclusions

While the molecular basis behind microbial lipid production under nitrogen-limitation has been explored over the years, there was no system study on the mechanism of Pi-limitation-induced lipid accumulation. Here, we prepared samples of *R. toruloides* cells in triplicates under Pi-limited chemostat conditions, and performed transcriptomic, proteomic, and metabolomic analysis. These multi-omic data were compared with those under Pi-replete conditions and integrated to reveal mechanistic insights. As a result, a brief picture was drawn to elaborate how cells respond to Pi-limitation and accumulate lipids (Fig. 1d). Major changes at different levels were identified along two apparently separated biological processes, namely, Pi-related metabolism and lipid accumulation, while key crosstalk between the two processes was also suggested.

First, Pi-limitation leads to Pi starvation in the cytosol, which triggers a global regulator of nutrient signaling. The CDK inhibitor Pho81 was up-regulated, leading to activation of the PHO pathway by up-regulating cyclin-dependent protein kinase complex Pho80–Pho85, high-affinity Pi transporters Pho84 and Pho89, as well as secretory phosphatases Pho5 (Fig. 1c). The high-affinity transporters could facilitate better Pi uptake, while the secretory phosphatases help Pi scavenging by cleaving phosphate ester bonds from different phosphorylated substrates including nucleic acids and phospholipids [47, 48]. In addition, there were other paths including degradation of polyphosphate and nucleotides being activated to liberate Pi. Thus, it was clear that cells reached a state with improved ability for Pi uptake and recycle under Pi-limited conditions [14, 48].

Second, Pi-limitation leads to activation of RNA degradation and inhibition of ribosome biosynthesis. This was a direct result of up-regulation of RNase Rny1 and down-regulation of many genes related to ribonucleoprotein biogenesis, respectively. Metabolomic analysis indicated that the contents of several nucleosides, bases, and their derivatives were significantly higher in cells cultivated under Pi-limitation (Additional file 7: Figure S1c). It should be mentioned that about 80% of total RNA is within ribosome located in the cytoplasm, and that cellular ribosome contents correlate closely with cell growth rate [49]. Thus, RNA degradation should result in reduced cell proliferation. As RNA degradation has neither been observed in oleaginous yeasts under nitrogen-limitation [5, 50], nor been documented for a number of microorganisms under Pi-limitation [15, 51–53], it thus turned out to be a unique biological feature awaiting further study.

Third, our data suggest that lipid accumulation under Pi-limitation is facilitated by several cellular process changes. The lipid droplet protein Ldp1 showed considerably higher levels in the Pi-limited sample, providing structural constituents to embrace more lipids and protect lipids from degradation. Higher levels of DAG were expected due to up-regulation of phosphatidate phosphatase Pah1 and down-regulation of DAG kinase Dgk1. The protein level of Acl1, Acs1, and Pdc1 were notably higher in the Pi-limited sample, indicating that more acetyl-CoA was produced from citrate, pyruvate, and acetate, respectively; while the protein level of Lys20 was remarkably lower, indicating that less acetyl-CoA was consumed for production of other metabolites. These results showed increased the formation of precursors at the early stage and late stage of TAG biosynthesis. In addition, up-regulation of the autophagy process could degrade some cellular organelles and provide more space and metabolites for lipid biosynthesis.

Fourth, our data suggest that nucleotide monophosphates’ (NMP) degradation mediated by phosphatases is one of the key metabolic events to establish molecular connection between Pi-limitation and lipid accumulation. The up-regulation of phosphatases Pho8 and Phm8 led to reduced contents of nucleotides but higher contents of nucleosides. Our biochemical data and previous studies [54, 55] indicate that one particular nucleotide, AMP, is an allosteric activator of Idh of oleaginous yeasts. Thus, down-regulation of Idh activity by reduced AMP level led to accumulation of citrate, which was readily converted into acetyl-CoA by Acl1.

**Fig. 4:**
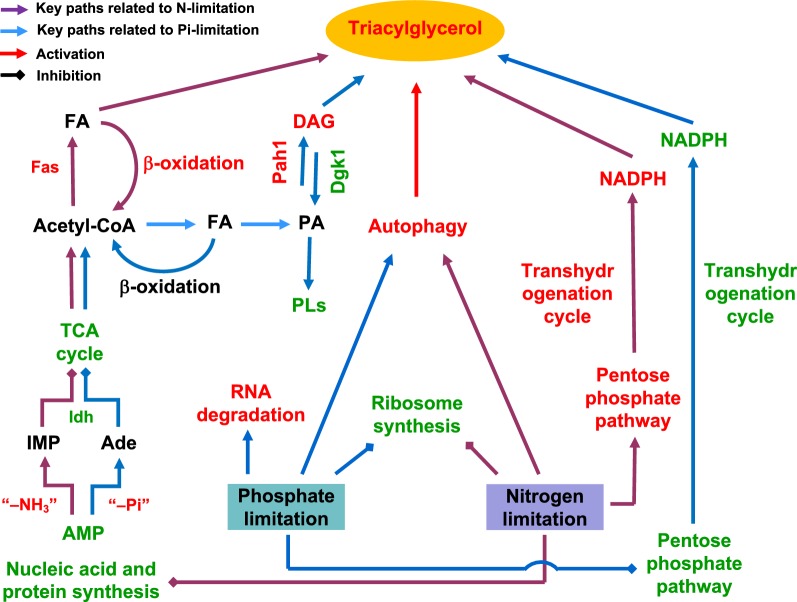
Global differences of cellular responses to Pi-limitation and nitrogen-limitation and lipid accumulation by *R. toruloides*. The red indicated activated process, up-regulated protein, or enriched metabolite, and the green indicated inhibited process, down-regulated protein, or lowered metabolite

Finally, Pi-limitation induced lipid accumulation is mechanistically distinguished in several aspects from the nitrogen-limitation induced process (Fig. 4). Under Pi-limitation, reduced cell proliferation is mainly resulted from RNA degradation and down-regulation of ribosome biogenesis; however, under nitrogen-limitation, cell proliferation is repressed due to down-regulation of biosynthesis of nucleic acids and protein. While TCA cycle activity is reduced due to allosteric down-regulation of Idh by lowered AMP levels, AMP is converted into adenosine (Ade) upon dephosphorylation under Pi-limitation but inosine monophosphate (IMP) upon deamination under nitrogen-limitation. Much lower NADPH level was observed due to reduced flux through the PP pathway and the Me-mediated transhydrogenation cycle together with enhanced NADPH consumption for lipid biosynthesis. Remarkably, under nitrogen-limitation, NADPH was in excess, and the transhydrogenation cycle was notably up-regulated. An early modeling study suggested that the PP pathway provides 63% of the required NADPH for lipid production on glucose, and the transhydrogenation cycle contributes a significant portion. In addition, the NADPH supply system is depending on different substrates [56]. While over-expression of Me1 in *R. toruloides* drastically enhanced lipid production on glucose under Pi-limited condition in this study, only minor lipid titer increase was observed in low-nitrogen media [46]. These differences are a striking reminder that the performances of engineered strains are ultimately highly contingent on the culture conditions.

Although we tried our efforts to line up evidences and build mechanistic insights, it should be emphasized that much information embedded in these omic data sets remains to be deconvoluted and verified in the future. For example, the connections among Pi-limitation, autophagy, and lipid accumulation are an interesting topic for in-depth analysis. It is also worthwhile studying those transcription factors being up- and down-regulated under Pi-limitation. Nonetheless, we have already been guided to engineer *R. toruloides* strain over-expressing malic enzyme, and indeed, the engineered strain produced more lipids than the wild-type strain did under Pi-limited conditions.

In summary, we have acquired multi-omic data to demonstrate that Pi-limitation facilitates up-regulation of Pi-related metabolism, RNA degradation, and TAG biosynthesis, while down-regulation of ribosome biosynthesis and TCA cycle, leading to enhanced carbon fluxes into lipids. This study greatly enriched our understanding on microbial oleaginicity, cellular responses to Pi-limitation, and their crosstalk. As products derived from the fatty acid biosynthetic pathway remain intensively pursued targets [2, 22, 23, 57] and genetic tools for *R. toruloides* are readily available [26, 42, 58, 59], we expect that the knowledge and data should help designing advanced cell factories for the production of various lipid derivatives.

## Additional files

**Additional file 1: Table S1.** Primers used in this study.

**Additional file 2: Table S2.** Composition of total raw reads and statistical results of samples mapped to gene and genome.

**Additional file 3: Table S3.** Differentially expressed genes between “P0” and “F3”.

**Additional file 4: Table S4.** Differentially expressed proteins between “P0” and “F3”.

**Additional file 5: Table S5.** Model summaries for the discrimination between the Pi-replete and Pi-limited samples from LC-MS data for metabolomic analysis.

**Additional file 6: Table S6.** Metabolites detected in sample “P0” and “F3”.

**Additional file 7: Figure S1.** Comparative metabolomic analyses of *R. toruloides* samples prepared under Pi-limited (P0) and Pi-replete (F3) conditions. **a** Total ion chromatogram of quality control (QC) sample (positive). **b** Total ion chromatogram of QC sample (negative). **c** Changes of some cellular nucleoside derivatives and bases. **Figure S2.** Metabolomic analysis of *R. toruloides* during phosphate-limitation. **a** Score plot of PCA in P0 vs F3 (positive). **b** Score plot of PLS-DA in P0 vs F3 (positive). **c** Sorting plot of PLS-DA in P0 vs F3 (positive). **d** Score plot of OPLS-DA in P0 vs F3 (positive). **Figure S3.** Metabolomic analysis of *R. toruloides* during phosphate-limitation. **a** Score plot of PCA in P0 vs F3 (negative). **b** Score plot of PLS-DA in P0 vs F3 (negative). **c** Sorting plot of PLS-DA in P0 vs F3 (negative). **d** Score plot of OPLS-DA in P0 vs F3 (negative).

## Abbreviations

Acc: acetyl-CoA carboxylase
Acl: ATP-citrate lyase
Acs: acetyl-CoA synthase
AMP: adenosine monophosphate
CDK: cyclin-dependent kinase
DAG: diacylglycerol
DCW: dry cell weight
DEGs: differentially expressed genes
DGK: diacylglycerol kinase
FA: fatty acids
Fas: fatty acid synthetase
FDR: false discovery rate
GO: gene ontology
Hcd: 3-hydroxyacyl-CoA dehyrogenase
Idh: isocitrate dehydrogenase
iTRAQ: isobaric tags for relative and absolute quantitation
LD: lipid droplets
Me: malic enzyme
NMP: nucleotide monophosphates
OPLS-DA: orthogonal partial least-squares discriminant analysis
Pah: phosphatidate phosphatase
PCA: principal components analysis
Pdc: pyruvate decarboxylase
Pi: inorganic phosphate
PLS-DA: partial least-squares-discriminant analysis
polyP: polyphosphate
PP: pentose phosphate
Pyc: pyruvate carboxylase
RPKM: reads per kilo bases per million reads
TAG: triacylglycerols
TCA: tricarboxylic acid
TIC: total ion chromatogram
