# Supplementary material for: Systems analysis of phosphate-limitation-induced lipid accumulation by the oleaginous yeast Rhodosporidium toruloides
Source: Biotechnol Biofuels. 2018 May 25;11:148. doi: 10.1186/s13068-018-1134-8 (PMC5968551; doi:10.1186/s13068-018-1134-8)
Supplement: Supplementary file 1 — Additional file 1: Table S1. Primers used in this study. [file 13068_2018_1134_MOESM1_ESM.doc]

**Table S1. Primers used in this study.**

| Primer | Sequence (5’–3’) | Description |
| --- | --- | --- |
| RHTO_00229-F | gactacaacgacatccacgaacc | Amplification of RHTO_00229 gene by RT-qPCR |
| RHTO_00229-R | cgaacgactctcctcaaacca |
| RHTO_03172-F | tgttgacggaggacgagtg | amplification of RHTO_03172 gene by RT-qPCR |
| RHTO_03172-R | aagaggaggatgtgcggttc |
| RHTO_04266-F | gttgatcttcgcctcgtgct | amplification of RHTO_04266 gene by RT-qPCR |
| RHTO_04266-R | ggggtcggtgatgtaaggaa |
| RHTO_07849-F | ccatgctgtccgagtattgaag | amplification of RHTO_07849 gene by RT-qPCR |
| RHTO_07849-R | gacgaagtagaggcgggaga |
| RHTO_01341 -F | ccgcattcgtcatctgtctc | amplification of RHTO_01341 gene by RT-qPCR |
| RHTO_01341-R | tgtcccaccacctgtttcc |
| RHTO_04309-F | acttcccgacccgatttctt | amplification of RHTO_04309 gene by RT-qPCR |
| RHTO_04309-R | ggatggtgtggatgaggtgtt |
| RHTO_05934-F | tcacgtcaaagaagcgatcaa | amplification of RHTO_05934 gene by RT-qPCR |
| RHTO_05934-R | ataatgtcaccgccccaga |
| RHTO_04894-F | aagtgacaacggcgaagga | mplification of RHTO_04894 gene by RT-qPCR |
| RHTO_04894-R | tagcgagacgagttgggagag |
| RHTO_06970-F | gttcgagagggcgtatgagg | mplification of RHTO_06970 gene by RT-qPCR |
| RHTO_06970-R | aggatagaacgagaggcaccaa |
| ACTIN-F | GCTGTCTTCCCCTCGATTGT | amplification of *ACTIN* gene by RT-qPCR |
| ACTIN-R | GGGTCAGGATACCACGCTTC |
| GAPDH-F | GGTATCGCCCTCAACGACA | amplification of *GAPDH* gene by RT-qPCR |
| GAPDH-R | GACGAGCAAGTCCACGACAC |
