# Supplementary material for: Systems analysis of phosphate-limitation-induced lipid accumulation by the oleaginous yeast Rhodosporidium toruloides
Source: Biotechnol Biofuels. 2018 May 25;11:148. doi: 10.1186/s13068-018-1134-8 (PMC5968551; doi:10.1186/s13068-018-1134-8)
Supplement: Supplementary file 2 — Additional file 2: Table S2. Composition of total raw reads and statistical results of samples mapped to gene and genome. [file 13068_2018_1134_MOESM2_ESM.doc]

**Table S2.** Composition of total raw reads and statistical results of samples mapped to gene and genome.

| Composition of total raw reads | | | | | | | | |
| --- | --- | --- | --- | --- | --- | --- | --- | --- |
| Sample ID | Clean reads | | Reads containing adaptor | | Reads containing N | | Reads of low quality | |
| P0 | 10479872 (97.47%) | | 168523 (1.57%) | | 0 (0.00%) | | 103894 (0.97%) | |
| F3 | 11590655 (98.13%) | | 115295 (0.98%) | | 0 (0.00%) | | 105417 (0.89%) | |
| Statistical results of samples mapped to gene | | | | | | | | |
|  | Total Reads | Total Base Pairs | Total Mapped Reads | Perfect Match | <= 2 bp Mismatch | Unique Match | Multi-position Match | Total Unmapped Reads |
| P0 | 10,479,872  (100.00%) | 513,510,726  (100.00%) | 5,703,512  (54.42%) | 4,259,737  (40.65%) | 1,443,775  (13.76%) | 5,641,377  (53.83%) | 62,135  (0.59%) | 4,776,360  (45.56%) |
| F3 | 11,590,655  (100.00%) | 567,912,095  (100.00%) | 5,953,147  (51.36%) | 1,162,853  (38.50%) | 1,490,291  (12.86%) | 5,892,590  (50.81%) | 60.557  (0.52%) | 5,637,508  (48.64%) |
| Statistical results of samples mapped to genome | | | | | | | | |
| P0 | 10,479,872  (100.00%) | 513,510,726  (100.00%) | 5,947,829  (56.75%) | 4,216,618  (40.24%) | 1,731,011  (16.52%) | 5,867,083  (55.98%) | 80,746  (0.77%) | 4,532,043  (43.25%) |
| F3 | 11,590,655  (100.00%) | 567,912,095  (100.00%) | 6,158,135  (53.13%) | 4,376,880  (37.76%) | 1,781,256  (15.37%) | 6,083,048  (52.48%) | 75.088  (0.65%) | 5,132,519  (16.87%) |
