# Supplementary material for: Systems analysis of phosphate-limitation-induced lipid accumulation by the oleaginous yeast Rhodosporidium toruloides
Source: Biotechnol Biofuels. 2018 May 25;11:148. doi: 10.1186/s13068-018-1134-8 (PMC5968551; doi:10.1186/s13068-018-1134-8)
Supplement: Supplementary file 5 — Additional file 5: Table S5. Model summaries for the discrimination between the Pi-replete and Pi-limited samples from LC-MS data for metabolomic analysis. [file 13068_2018_1134_MOESM5_ESM.doc]

**Table S5. Model summaries for the discrimination between the Pi-replete and Pi-limited samples from LC-MS data for metabolomic analysis**

|  | Positive ion model | | | Negative ion model | | |
| --- | --- | --- | --- | --- | --- | --- |
|  | R^2^X | R^2^Y | Q^2^ | R^2^X | R^2^Y | Q^2^ |
| PCA | 0.588 | - | 0.272 | 0.671 | - | 0.330 |
| PLS-DA | 0.478 | 0.999 | 0.975 | 0.434 | 0.966 | 0.893 |
| OPLS-DA | 0.571 | 1.000 | 0.980 | 0.643 | 0.990 | 0.948 |
