# Supplementary material for: Systems analysis of phosphate-limitation-induced lipid accumulation by the oleaginous yeast Rhodosporidium toruloides
Source: Biotechnol Biofuels. 2018 May 25;11:148. doi: 10.1186/s13068-018-1134-8 (PMC5968551; doi:10.1186/s13068-018-1134-8)
Supplement: Supplementary file 7 — Additional file 7: Figure S1. Comparative metabolomic analyses of R. toruloides samples prepared under Pi-limited (P0) and Pi-replete (F3) conditions. a Total ion chromatogram of quality control (QC) sample (positive). b Total ion chromatogram of QC sample (negative). c Changes of some cellular nucleoside derivatives and bases. Figure S2. Metabolomic analysis of R. toruloides during phosphate-limitation. a Score plot of PCA in P0 vs F3 (positive). b Score plot of PLS-DA in P0 vs F3 (positive). c Sorting plot of PLS-DA in P0 vs F3 (positive). d Score plot of OPLS-DA in P0 vs F3 (positive). Figure S3. Metabolomic analysis of R. toruloides during phosphate-limitation. a Score plot of PCA in P0 vs F3 (negative). b Score plot of PLS-DA in P0 vs F3 (negative). c Sorting plot of PLS-DA in P0 vs F3 (negative). d Score plot of OPLS-DA in P0 vs F3 (negative). [file 13068_2018_1134_MOESM7_ESM.pdf]

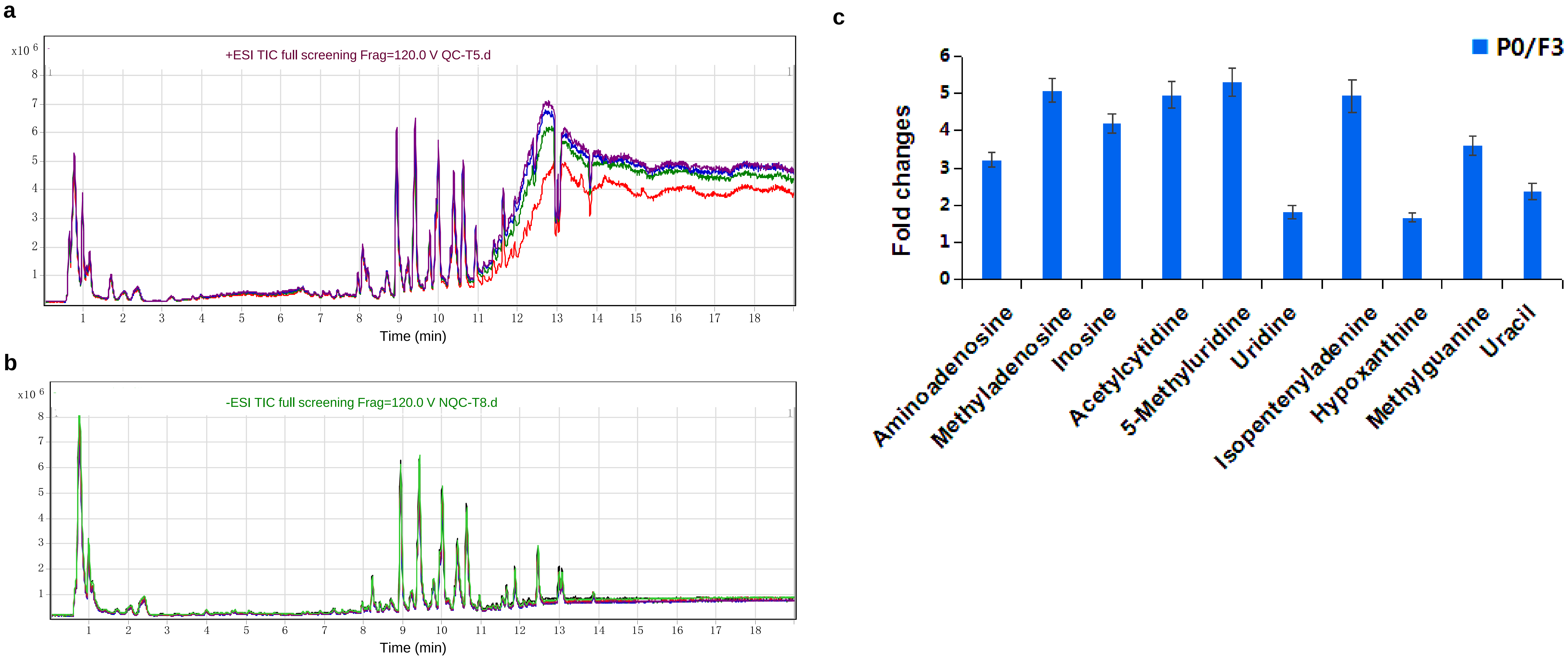

Figure S1. Comparative metabolomic analyses of *R. toruloides* samples prepared under Pi-limited (P0) and Pi-replete (F3) conditions. **a** Total ion chromatogram of quality control (QC) sample (positive). **b** Total ion chromatogram of QC sample (negative). **c** Changes of some cellular nucleoside derivatives and bases.

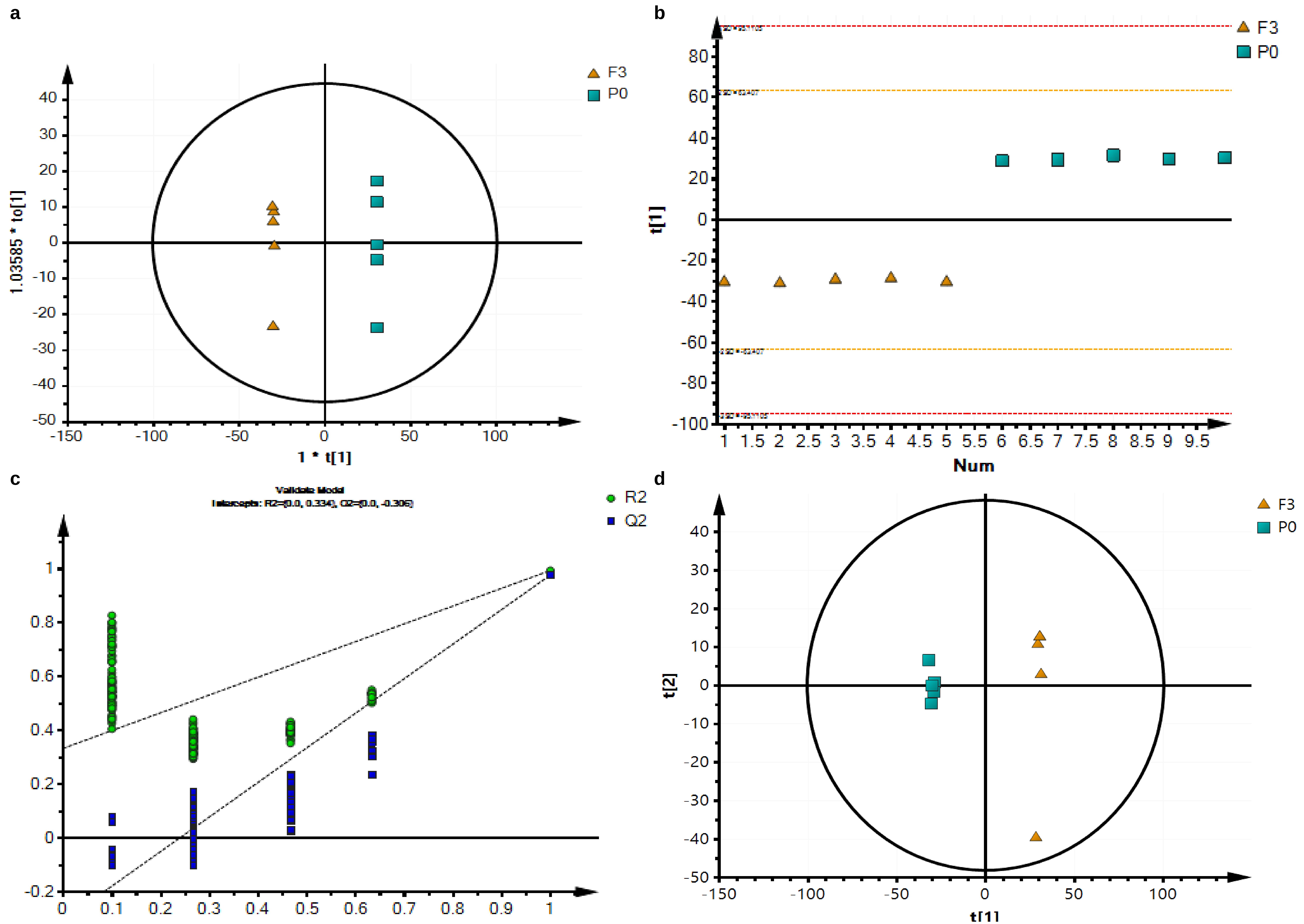

Figure S2. Metabolomic analysis of *R. toruloides* during phosphate limitation. **a** The score plot of PCA in P0 vs F3 (positive). **b** The score plot of PLS-DA in P0 vs F3 (positive). **c** The sorting plot of PLS-DA in P0 vs F3 (positive). **d** The score plot of OPLS-DA in P0 vs F3 (positive).

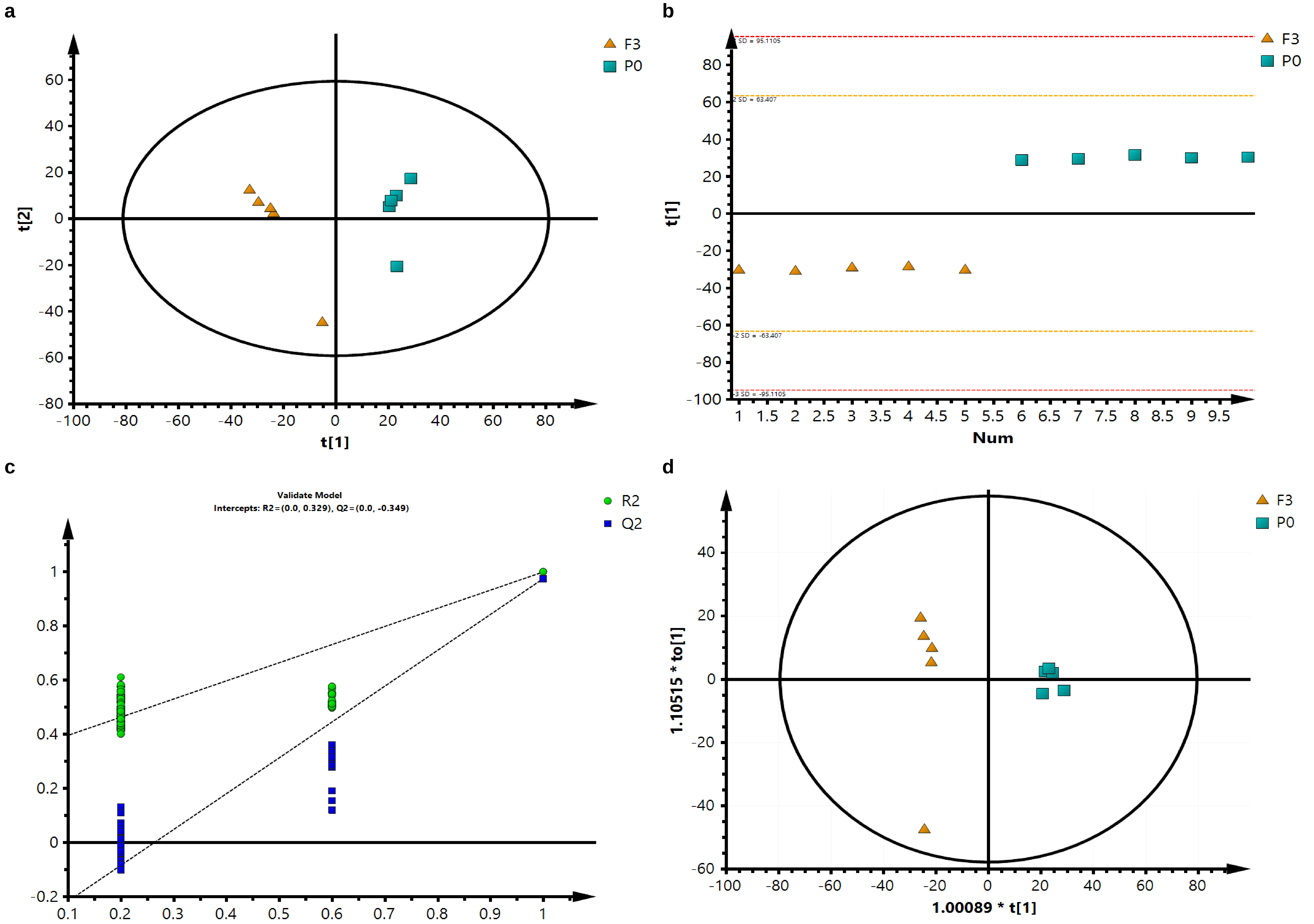

Figure S3. Metabolomic analysis of *R. toruloides* during phosphate limitation. **a** The score plot of PCA in P0 vs F3 (negative). **b** The score plot of PLS-DA in P0 vs F3 (negative). **c** The sorting plot of PLS-DA in P0 vs F3 (negative). **d** The score plot of OPLS-DA in P0 vs F3 (negative).
